# Thrombotic thrombocytopenic purpura in a patient on long-term alpha-interferon therapy for essential thrombocythemia: a case report

**DOI:** 10.1186/s12882-023-03200-7

**Published:** 2023-05-23

**Authors:** Chunmei Qin, Dan Yin, Fang Liu, Hongyu Qiu

**Affiliations:** 1 Department of Nephrology, Kidney Research Institute, West China Hospital of Sichuan University, 37, Guoxue Alley, Chengdu, 610041 Sichuan Province China; 2 Department of Nephrology, Luzhou People’s Hospital, Luzhou, China; 3 Bioinformatics Under Biology Department, University of California-San Diego, San Diego, USA

**Keywords:** Essential thrombocythemia, Thrombotic thrombocytopenic purpura, Alpha interferon, Plasma exchange, Case report

## Abstract

**Background:**

Thrombotic thrombocytopenic purpura (TTP) is rare and severe thrombotic microangiopathy characterized by thrombocytopenia, hemolytic anemia, and renal dysfunction. In contrast, essential thrombocythemia (ET) is a myeloproliferative disease associated with an abnormal increase in platelet numbers. Previous studies reported several cases of the development of ET in patients with TTP. However, the case of an ET patient complicated with TTP has not been previously reported. In this case study, we present a patient with TTP who was previously diagnosed with ET. Therefore, to the best of our knowledge, this is the first report of TTP in ET.

**Case presentation:**

A 31-year-old Chinese female who was previously diagnosed with ET presented with anemia and renal dysfunction. The patient had been on long-term treatment with hydroxyurea, aspirin, and alpha interferon (INF-α) for ten years. The diagnosis of TTP was confirmed by clinical features, schistocytes noted on the peripheral blood smear, and lower ADAMTS13 activity (8.5%), together with the renal biopsy results. INF-α was discontinued, and the patient was then treated with plasma exchange and corticosteroids. After one year of follow-up, the patient had a normal hemoglobin level and platelet numbers, and her ADAMTS13 activity had improved. However, the patient’s renal function remains impaired.

**Conclusions:**

We report a case of an ET patient complicated with TTP that was possibly due to INF-α, highlighting the potential complications associated with long-term ET therapy. The case also highlights the importance of considering TTP in patients with pre-existing ET who present with anemia and renal dysfunction, extending the spectrum of known studies.

## Background

Thrombotic thrombocytopenic purpura (TTP) and essential thrombocythemia (ET) are two hematologic disorders characterized by apparently opposite clinical features. TTP is a rare and severe life-threatening disease commonly defined by thrombocytopenia (platelet count less than 100×10^9^/L), microangiopathic hemolytic anemia (MAHA), neurologic changes, renal dysfunction, and fever [1]. It is caused by severely reduced activity of the von Willebrand factor-cleaving protease ADAMTS13 (a disintegrin and metalloproteinase with thrombospondin motifs 13). In contrast, ET was classified as a myeloproliferative neoplasm by Damesheck in 1951. According to the World Health Organization, ET is identified by a sustained elevation in platelet numbers (more than 450×10^9^/L) and megakaryocytic hyperplasia in the bone marrow [2]. Driver mutations, such as JAK2V617F (55%), CALR (20%-25%), and MPL (5%), are also often associated [3]. The primary complications in ET are thrombohemorrhagic events and leukemic/fibrotic transformation. Renal involvement by ET is rare. However, renal failure may develop in patients with ET due to bilateral thrombosis of the renal arteries or occlusion of the urinary tract by blood clots [4, 5]. Here, we report a patient with ET who developed anemia and renal dysfunction after treatment with alpha interferon (INF-α).

## Case presentation

A 31-year-old female patient was admitted to the Department of Nephrology at West China Hospital after experiencing edema and fatigue for approximately seven days. She did not have a fever, diarrhea, purpura, or dark urine. The patient had a ten-year history of ET and was prescribed hydroxyurea, aspirin, and INF-α. The patient received a dose of interferon of 3×10^6^ IU/week for the past five years and received the latest interferon treatment four days before arriving at our unit. The patient’s blood pressure and blood tests were monitored every three months throughout her interferon treatment, including liver enzymes, complete blood count, and renal function, none of which were abnormal. Her personal, family, and psychosocial histories were unremarkable. Upon physical examination, her temperature was 36.5 ℃, heart rate was 75 beats per minute, respiratory rate was 20 breaths per minute, and blood pressure was 140/90 mmHg. The patient’s weight was 55 kg, and her height was 158 cm. No skin rash or bleeding spots were seen, but moderate edema was observed on the face and extremities. The muscle strength and muscle tone of the four limbs were normal, and no abnormalities were identified in the physical examination of the lung, heart, and abdomen. Laboratory test results revealed anemia with schistocytosis, elevated lactic dehydrogenase (LDH) and creatinine levels, and a negative Coomb's tests, raising concern for thrombotic microangiopathy (TMA) (Table 1). Thus, hydroxyurea and INF-α were discontinued, and prednisone at 1 mg/kg/day was started.

**Table 1:** Biochemical characteristics of the patient

Examination item	Test value	Reference value
Hemoglobin (g/L)	69	115–150
Platelet (10^9^/L)	205	100–300
White blood cell (10^9^/L)	5.91	3.5–9.5
Albumin (g/L)	26	40.0–55.0
Glucose (mmol/L)	4.78	3.90–5.90
Creatinine (umol/L)	388	48–79
BUN (mmol/L)	14.3	2.6–7.5
eGFR (ml/min/1.73m^2^)	12.59	56–122
Uric Acid (umol/L)	527	160–380
Triacylglycerol (mmol/L)	1.98	0.29–1.83
Cholesterol (mmol/L)	5.5	2.80–5.70
Lactate dehydrogenase (IU/L)	893	120–250
Schistocytes (%)	5	< 1
ADAMTS13 activity (%)	8.5	68–131
24-h Urine protein (g/24 h)	8.13	< 0.15
Urine protein creatinine ratio (g/mmol Cr)	1.704	< 0.045
Antinuclear antibody	Negative	Negative
Anti-double-stranded DNA antibody (IU/ml)	Negative	< 30
Anti-Sm antibody	Negative	Negative
Coomb's tests	Negative	Negative
Complement C3 (g/L)	1.03	0.785–1.520
Complement C4 (g/L)	0.352	0.145–0.360
Antineutrophil cytoplasmic antibodies	Negative	Negative
Anti-glomerular basement membrane antibody	Negative	Negative

**Fig. 1:**
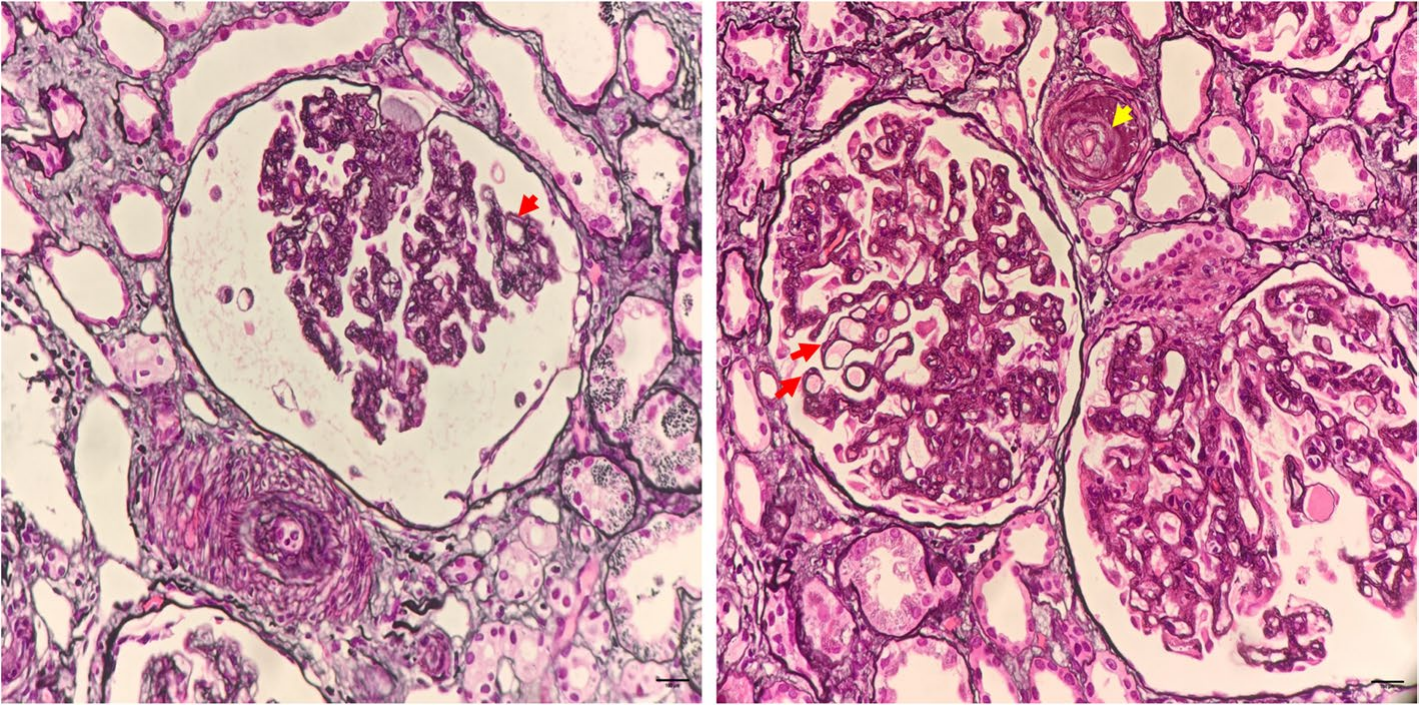
Characteristic histological findings in the renal biopsy. Periodic acid-methylamine silver staining shows a double-contour appearance of the basement membrane (red arrows) and an arteriole with luminal narrowing due to intimal edema and widening (yellow arrow)

A central venous catheter was placed due to a further increase in creatinine (529 umol/L) and oliguria, and hemodialysis combined with single-volume plasma exchange (PE) was conducted after obtaining a blood sample for the ADAMTS 13 activity test. Given the patient’s history of ET, leukemic or fibrotic disease transformation was a leading consideration in this case. Autoimmune diseases and infections were also suspected as possible causes. However, the bone marrow biopsy showed proliferation mainly of megakaryocyte lineage cells, with increased numbers of enlarged, mature megakaryocytes with hyper-lobulated nuclei. Tests for tumor markers, antinuclear antibody, extractable nuclear antigens, anticardiolipin antibodies, antineutrophil cytoplasmic autoantibody, complement C3, complement C4, complement factor B, human immunodeficiency virus antibody test, hepatitis B virus DNA, Epstein-Barr virus DNA, and septic work-up, including blood, urine, and stool, were negative. The coagulation assay and fibrinogen test results were also within the normal ranges. We did not observe any typical findings of malignant hypertension on her optic fundi. The patient’s ADAMTS 13 activity was 8.5%, and brain computed tomography scan and ultrasound examinations of the kidneys did not reveal any abnormalities. The renal biopsy showed a double contour of the basement membrane and arteriole with luminal narrowing. Immunofluorescence tests for IgG, IgA, IgM, C3, C4, and C1q were all negative. The electron micrograph showed diffuse endothelial swelling with obliteration of the capillary lumina. A diagnosis of TMA was made based on the pathological examination (Fig. 1).

**Fig. 2:**
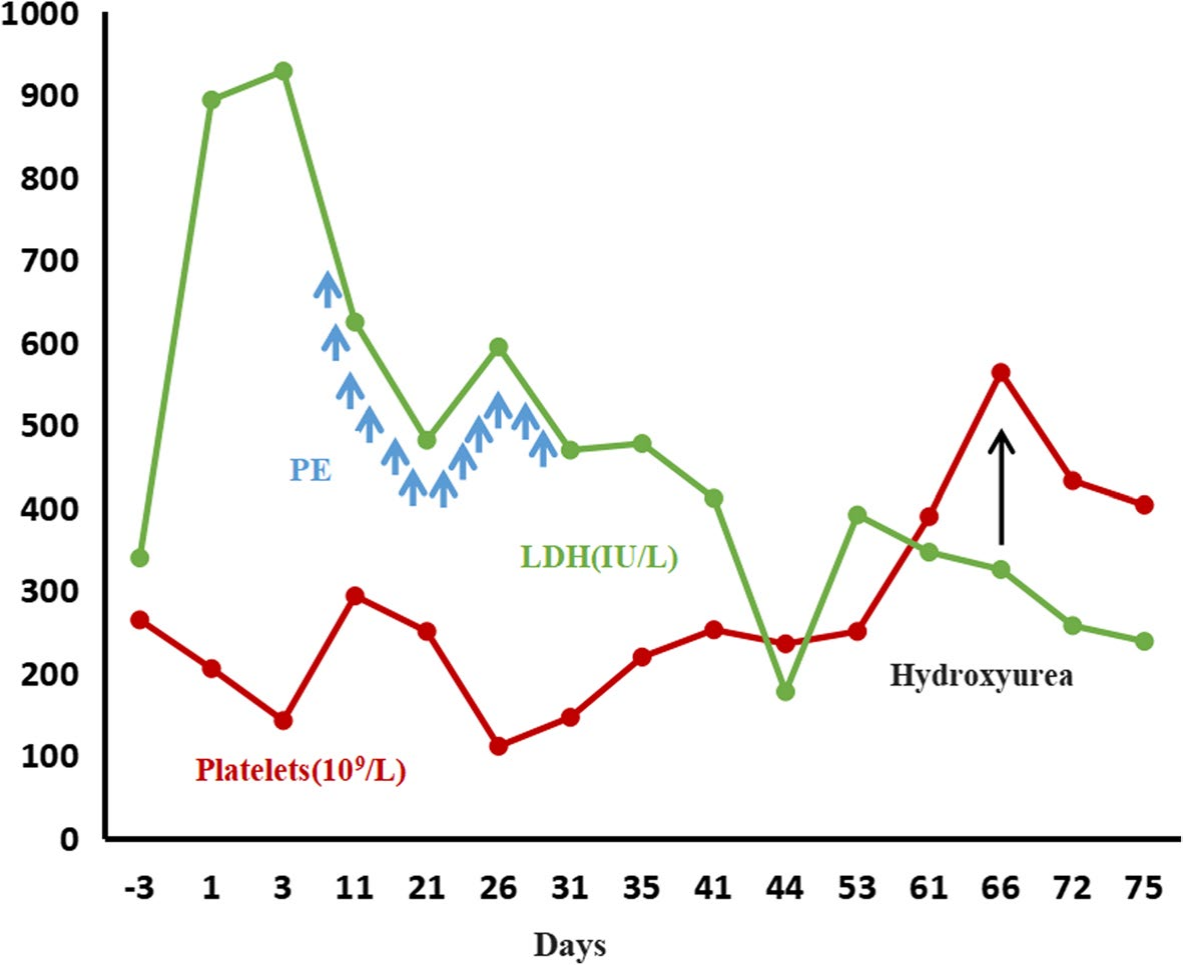
Dynamic changes in platelet counts and lactate dehydrogenase (LDH) levels during hospitalization. The plasma exchange (PE) dates are denoted by blue arrows and hydroxyurea administration is denoted by a thin, black arrow

Following five cycles of PE and hemodialysis, the patient exhibited improved clinical symptoms, including elevated hemoglobin level, normalized platelet count, and decreased LDH level (Fig. 2). PE was then paused after 12 cycles. By day 66, the repeated ADAMTS 13 test showed that the activity had increased to 35.8%, and the platelet count had risen to 563×10^9^/L. Then, hydroxyurea was added to the treatment regimen. On day 75, the patient was discharged in stable condition. Unfortunately, despite the stable results, renal function had not recovered, and she still required continuous hemodialysis during her one-year follow-up.

## Discussion and conclusions

As ET and TTP have distinct natural histories and require different treatment modalities, it is crucial to acknowledge that the two diseases may present sequentially. A published case described the development of ET in a patient with TTP [6]. However, there have been no reported cases of ET patients complicated with TTP. To our knowledge, this was the first documented report of a patient with both ET and TTP. This case highlights the complexity and variability of platelet disorders and emphasizes the importance of reviewing peripheral blood smears, ADAMTS13 activity to evaluate TTP, and bone marrow aspirations to diagnose thrombocythemia accurately.

In our case, the renal biopsy revealed TMA, which commonly encompasses hemolytic uremia syndrome (HUS) and TTP. Typical HUS accounts for 90% of HUS cases and is caused by Shiga toxin-producing organisms *Escherichia coli* and *Shigella dysenteriae* [7]. Typical HUS predominantly affects children and has a classic prodrome of abdominal pain with diarrhea. In contrast, atypical HUS, which lacks prodromal symptoms, is caused by complement dysregulation and can be triggered by an upper respiratory tract infection [8]. Our patient was an adult with no prodrome of abdominal pain or diarrhea, and no significant abnormality was found in complement tests or fecal culture, reducing the possibility of HUS.

TTP, first described by Moschcowitz in 1924, is a life-threatening form of TMA characterized by systemic microvascular platelet aggregation and erythrocyte destruction. Previous studies showed that ADAMTS13 activity below 10% was specific for TTP and could be used to differentiate TTP from HUS and other TMAs [9]. The incidence of TTP is 2.2/1,000,000 per year, with a prevalence of 13-20/1,000,000, and is more common in women [10, 11]. Untreated TTP has a high mortality rate. However, with proper treatment, the 30-day mortality rate can be reduced to 4 – 7% [12]. Some direct deaths can be attributed to delayed diagnosis and, thus, postponed treatment [13]. Given the risk of acute disseminated microthrombus formation, irreversible organ damage, and death, it is critical to initiate treatment in patients with suspected TTP. PE is initiated once the diagnosis of TTP is suspected, acting as the cornerstone of immediate treatment [14, 15]. In our case, the patient presented with two symptoms of classic TTP pentalogy (MAHA and renal failure) and an ADAMTS13 activity of 8.5%. Therefore, after discontinuing INF-α and hydroxyurea, we immediately treated the patient with PE and corticosteroid (1 mg/kg/d).

TTP could be caused by a variety of factors, including infection, cancer, autoimmune diseases (such as systemic lupus erythematous, Gougerot-Sjögren syndrome, and antiphospholipid syndrome), organ transplantation, pregnancy, and drugs [1]. In this particular case, the patient had ET, but her bone marrow histopathology showed a stable condition without signs of leukemic or fibrotic disease transformation. The patient also did not exhibit any symptoms of autoimmune diseases (arthralgia or rash), and the related laboratory tests (antinuclear antibody, anticardiolipin antibody, antineutrophil cytoplasmic autoantibody, and Coomb's test) were all negative. The patient showed no signs of infection. Therefore, drug-induced TTP was suspected based on these factors.

Drug-induced TTP has been recognized in the last few decades, accounting for about 12% of TTP cases [16]. However, the majority of drug-induced TTP case reports are challenging to interpret because of the ambiguity surrounding the relationship between drug exposure and the onset of TTP [17]. The most common drugs associated with TTP are ticlopidine, quinine, clopidogrel (immune-mediated reaction), mitomycin C, and cyclosporine (dose-related toxicity) [17]. In this report, our patient had been treated with hydroxyurea [18, 19] and INF-α [20], both of which have been reported to cause TTP. However, retreatment with hydroxyurea did not worsen the MAHA symptoms, and her condition became more stable. Therefore, it was improbable that hydroxyurea was the drug responsible for inducing TTP. A causality assessment using the Naranjo algorithm was also performed [21]. The patient scored 6 points on the algorithm for INF-α (a score of “probable” on the probability scale), indicating that INF-α was probably the drug causing TTP.

Due to its antineoplastic, antiviral, and immunomodulatory properties, INF-α has been extensively used in the treatment of various human diseases [22]. It is actively used in the treatment of hematological malignancies, such as myeloproliferative neoplasms [22]. INF-α also plays a prominent role in the treatment of viral syndromes, such as hepatitis C [23]. However, despite its beneficial therapeutic properties, INF-α therapy has been associated with several well-documented toxicities [24]. Previously published cases described TTP as a side effect of INF-α in patients with viral hepatitis, chronic myelogenous leukemia, and polycythemia vera [25–28]. These cases emphasized a causal relationship between INF-α therapy and TMA [20]. To our knowledge, TTP associated with INF-α has not been reported in patients with ET. The characteristics of TTP in our case were different from other TTP patients that used INF-α. In our case, the patient's platelet count was not significantly reduced (platelet count was not less than 100×10^9^/L), which may be related to the patient’s ET. Diagnosing TTP clinically was challenging as the patient's platelet level did not show a significant reduction. TTP also complicated the treatment of ET and required better doctor-patient coordination.

The primary treatment for ET aims to prevent thrombotic complications. Aspirin therapy or observation alone is effective for lower-risk patients, while cytoreductive therapy is reserved for patients with high-risk disease [29]. Moreover, when TTP occurs, cytoreductive therapy is discontinued, and PE and corticosteroid therapy are initiated immediately. Patients require long-term dynamic monitoring by peripheral blood smears, ADAMTS13 activity, and other tests so that doctors can adjust the treatment plan and maintain ET and TTP in a relatively stable state.

Drug-induced TTP can occur through various mechanisms, including toxic dose-related reactions, immune reactions, and metabolism-mediated mechanisms [30]. However, the exact mechanisms underlying INF-α-associated TTP remain unclear. Some studies suggested that INF-α could cause direct damage to endothelial cells by decreasing vascular endothelial growth factor production or generating an ADAMTS-13 inhibitor through T-cell activation [20, 31]. Recent research on TTP pathogenesis identified a common pathway of complement activation in all TTP patients [32]. Further studies are necessary to elucidate the mechanism of TTP induced by INF-α.

In conclusion, we report the case of a patient with ET who developed TTP that was possibly due to INF-α. PE might help to control disease activity, but further research is needed for a better understanding of the mechanisms underlying this rare complication and the future detection of TTP in a timely manner.

## Data Availability

All data used to support the findings of this study are included in the article.

## Abbreviations

TTP: Thrombotic thrombocytopenic purpura
ET: Essential thrombocythemia
MAHA: Microangiopathic hemolytic anemia
LDH: Lactic dehydrogenase
TMA: Thrombotic microangiopathy
PE: Plasma exchange
HUS: Hemolytic uremia syndrome
